# Risk Factors for Bleeding After Endoscopic Submucosal Dissection for Gastric Cancer in Elderly Patients Older Than 80 Years in Japan

**DOI:** 10.14309/ctg.0000000000000404

**Published:** 2021-09-24

**Authors:** Mitsushige Sugimoto, Waku Hatta, Yosuke Tsuji, Toshiyuki Yoshio, Yohei Yabuuchi, Shu Hoteya, Hisashi Doyama, Yasuaki Nagami, Takuto Hikichi, Masakuni Kobayashi, Yoshinori Morita, Tetsuya Sumiyoshi, Mikitaka Iguchi, Hideomi Tomida, Takuya Inoue, Tatsuya Mikami, Kenkei Hasatani, Jun Nishikawa, Tomoaki Matsumura, Hiroko Nebiki, Dai Nakamatsu, Ken Ohnita, Haruhisa Suzuki, Hiroya Ueyama, Yoshito Hayashi, Masaki Murata, Shinjiro Yamaguchi, Tomoki Michida, Tomoyuki Yada, Yoshiro Asahina, Toshiaki Narasaka, Shiko Kuribayashi, Shu Kiyotoki, Katsuhiro Mabe, Mitsuhiro Fujishiro, Atsushi Masamune, Takashi Kawai

**Affiliations:** 1 Department of Gastroenterological Endoscopy, Tokyo Medical University Hospital, Tokyo, Japan;; 2 Division of Digestive Endoscopy, Shiga University of Medical Science Hospital, Shiga, Japan;; 3 Division of Gastroenterology, Tohoku University Graduate School of Medicine, Sendai, Japan;; 4 Department of Gastroenterology, Graduate School of Medicine, The University of Tokyo, Tokyo, Japan;; 5 Department of Gastroenterology, Cancer Institute Hospital, Japanese Foundation for Cancer Research, Tokyo, Japan;; 6 Division of Endoscopy, Shizuoka Cancer Center, Shizuoka, Japan;; 7 Department of Gastroenterology, Toranomon Hospital, Tokyo, Japan;; 8 Department of Gastroenterology, Ishikawa Prefectural Central Hospital, Kanazawa, Japan;; 9 Department of Gastroenterology, Osaka City University Graduate School of Medicine, Osaka, Japan;; 10 Department of Endoscopy, Fukushima Medical University Hospital, Fukushima, Japan;; 11 Department of Endoscopy, The Jikei University School of Medicine, Tokyo, Japan;; 12 Department of Gastroenterology, Kobe University International Clinical Cancer Research Center, Kobe, Japan;; 13 Department of Gastroenterology, Kobe University Graduate School of Medicine, Kobe, Japan;; 14 Department of Gastroenterology, Tonan Hospital, Sapporo, Japan;; 15 Second Department of Internal Medicine, Wakayama Medical University, Wakayama, Japan;; 16 Gastroenterology Center, Ehime Prefectural Central Hospital, Matsuyama, Japan;; 17 Department of Gastroenterology and Metabology, Ehime University Graduate School of Medicine, Toon, Japan;; 18 Division of Gastroenterology and Hepatology, Osaka General Medical Center, Osaka, Japan;; 19 Division of Endoscopy, Hirosaki University Hospital, Hirosaki, Japan;; 20 Department of Gastroenterology, Fukui Prefectural Hospital, Fukui, Japan;; 21 Faculty of Laboratory Science, Yamaguchi University Graduate School of Medicine, Ube, Japan;; 22 Department of Gastroenterology, Chiba University Graduate School of Medicine, Chiba, Japan;; 23 Department of Gastroenterology, Osaka City General Hospital, Osaka, Japan;; 24 Department of Gastroenterology, Toyonaka Municipal Hospital, Toyonaka, Japan;; 25 Department of Gastroenterology and Hepatology, Nagasaki University Hospital, Nagasaki, Japan;; 26 Endoscopy Division, National Cancer Center Hospital, Tokyo, Japan;; 27 Department of Gastroenterology, Juntendo University School of Medicine, Tokyo, Japan;; 28 Department of Gastroenterology and Hepatology, Osaka University Graduate School of Medicine, Suita, Japan;; 29 Department of Gastroenterology, National Hospital Organization Kyoto Medical Center, Kyoto, Japan;; 30 Division of Gastroenterology, Kansai Rosai Hospital, Amagasaki, Japan;; 31 Department of Gastroenterology and Hepatology, Saitama Medical Center, Saitama, Japan;; 32 Gastrointestinal Oncology, Osaka International Cancer Institute, Osaka, Japan;; 33 Division of Gastroenterology and Hepatology, Kohnodai Hospital, National Center for Global Health and Medicine, Chiba, Japan;; 34 Department of Gastroenterology, Kanazawa University Hospital, Kanazawa, Japan;; 35 Division of Endoscopic Center, University of Tsukuba Hospital, Tsukuba, Japan;; 36 Department of Gastroenterology and Hepatology, Gunma University Graduate School of Medicine, Maebashi, Japan;; 37 Department of Gastroenterology, Shuto General Hospital, Yamaguchi, Japan;; 38 Department of Gastroenterology, National Hospital Organization Hakodate National Hospital, Hakodate, Japan;; 39 Department of Gastroenterology and Hepatology, Nagoya University Graduate School of Medicine, Nagoya, Japan.

## Abstract

**INTRODUCTION:**

As the aging of people in a society advances, the number of elderly patients older than 80 years in Japan with gastric cancer continues to increase. Although delayed ulcer bleeding is a major adverse event after endoscopic submucosal dissection (ESD), little is known about characteristic risk factors for bleeding in elderly patients undergoing ESD. This study aimed to evaluate risk factors for delayed bleeding after ESD for gastric cancer in elderly patients older than 80 years.

**METHODS:**

We retrospectively evaluated the incidence of delayed bleeding after ESD in 10,320 patients with early-stage gastric cancer resected by ESD between November 2013 and January 2016 at 33 Japanese institutions and investigated risk factors for delayed bleeding in elderly patients older than 80 years.

**RESULTS:**

The incidence of delayed bleeding in elderly patients older than 80 years was 5.7% (95% confidence interval [CI]: 4.6%–6.9%, 95/1,675), which was significantly higher than that in nonelderly (older than 20 years and younger than 80 years) patients (4.5%, 4.1%–5.0%, 393/8,645). Predictive factors for ESD-associated bleeding differed between nonelderly and elderly patients. On multivariate analysis of predictive factors at the time of treatment, risk factors in elderly patients were hemodialysis (odds ratio: 4.591, 95% CI: 2.056–10.248, *P* < 0.001) and warfarin use (odds ratio: 4.783, 95% CI: 1.689–13.540, *P* = 0.003).

**DISCUSSION:**

This multicenter study found that the incidence of delayed bleeding after ESD in Japanese patients older than 80 years was high, especially in patients receiving hemodialysis and taking warfarin. Management of ESD to prevent delayed bleeding requires particular care in patients older than 80 years.

## INTRODUCTION

Gastric cancer (GC), one of the major types of cancers, is particularly prevalent in East Asian countries such as Japan, Korea, and China. Indeed, against a world age-standardized incidence rate per 100,000 persons in 2012 of 12.1 (17.4 in men and 7.5 in women), the age-standardized incidence rate for Korea in 2018 was 39.6, followed by 27.5 in Japan, and 20.7 in China (1). Among treatments, endoscopic submucosal dissection (ESD) was developed as an endoscopic procedure for early-stage GC (2). ESD provides complete but minimally invasive pathological assessment and is generally selected as the first-line treatment for early-stage GC (2). However, several lesion-related factors can prolong procedure time. These include a large lesion size and the presence within the lesion of ulceration, scarring, and fibrosis. These in turn are considered to increase the risk of adverse events, such as delayed bleeding and perforation (3–5). Moreover, several procedure-related factors also influence procedure time and the incidence of adverse events, including type of knife and coagulation mode and endoscopist experience (3–7). Of the many possible factors affecting bleeding after ESD for GC, antithrombotic drugs are regarded as a major risk factor (8,9).

The proportion of the world's population older than 60 years will nearly double between 2015 and 2050, from 12% (900 million) to 22% (2 billion). The World Health Organization defines people aged 65–74 years as early-stage elderly population and those aged 75 years or older as late-stage elderly population. Over the past 2 decades, the elderly population of Japanese aged 85 years or older has increased from 1.4 to 4.8 million, or from 1.2% to 3.8% of the total (10). The number of elderly patients older than 80 years with GC in Japan has also increased. Elderly patients may be more likely to experience severe adverse events than nonelderly patients, including delayed bleeding after ESD because of physical weakness, poor general condition, comorbidities, and a generally higher intake of several drugs, including antithrombotic and nonsteroidal antiinflammatory agents (11). Accordingly, the efficacy and safety of ESD in elderly patients warrants particular consideration. Because elderly patients are generally at higher risk of multicausal death, the need for ESD in the elderly patients should consider both efficacy and safety and whether the risk factors of adverse events after ESD are similar to those of nonelderly patients.

In this study, we conducted a retrospective multicenter trial of Japanese elderly patients with early-stage GC treated at 33 Japanese institutions (12). This study was conducted as a subanalysis of our previous study (12) and had 2 goals: evaluating the incidence of ulcer bleeding after ESD for GC in elderly patients older than 80 years and clarifying risk factors of ESD-associated delayed bleeding at the time of treatment.

## METHODS

### Study design and patients

We retrospectively enrolled 11,452 patients who were scheduled to undergo ESD for early-stage GC at 33 Japanese institutions between November 2013 and October 2016 (12). Inclusion criteria were patient aged 20 years or older and early-stage GC clinically diagnosed. Patients were excluded if they failed to complete ESD endoscopically and pathologically, had a follow-up duration of <28 days after ESD, underwent closure of an ulcer after ESD, received polyglycolic acid (PGA) sheets and fibrin glue after ESD, received additional photodynamic therapy after ESD, refused the use of their clinical data, had invasion to the muscularis propria or deeper, or had remnant stomach. The criteria used to diagnose early-stage GC for ESD were consistent with Japanese GC treatment guidelines (13,14). Patients with intraoperative bleeding during the ESD procedure were included.

The study protocol conformed to the ethical guidelines of the Declaration of Helsinki and was reviewed and approved by the Institutional Review Board of each institution before recruitment. This study was conducted as a subanalysis of our previous study (12).

### ESD for GC

ESD for early-stage GC was performed based on a standard ESD procedure at all institutions. The type of endoscope, knife used as the cutting device, and electrosurgical generator used during the endoscopic treatment depended on the institution. A scheduled second-look endoscopic procedure also depended on the institution.

In patients taking antithrombotic drugs, most ESDs were performed according to guidelines for gastroenterological endoscopy in patients undergoing antithrombotic treatment published by the Japan Gastroenterological Endoscopy Society (15).

### Definition of bleeding

We defined post-ESD delayed bleeding as gastric bleeding with clinical signs (e.g., hematemesis, melena, and bloody stool) confirmed by emergency endoscopy within 28 days post-ESD. Clinical symptoms were defined as hematemesis, melena, or a decrease in hemoglobin of >2 g/dL since the most recent laboratory test.

### Statistical analysis

Values for age, hospital stay duration, and tumor size are given as the mean ± SD. Categorical variables were summarized as numbers and percentages, and statistically significant differences in category variables between the 2 groups (nonelderly patients older than 20 years and younger than 80 years vs elderly patients older than 80 years) were determined by the χ^2^ test. Statistically significant differences in mean values between the 2 groups were determined by the *t* test. Multivariate logistic regression analyses were used to test the associations of 16 candidate variables with bleeding after ESD for early-stage GC at the time of treatment. Multicollinearity among variables was tested using the variance inflation factor. *P* < 0.05 was considered statistically significant, and all *P* values were 2-sided. Calculations were conducted using SPSS version 26 (IBM, Armonk, NY). All authors had access to the study data and reviewed and approved the final manuscript.

## RESULTS

### Patient characteristics

**Figure 1.**
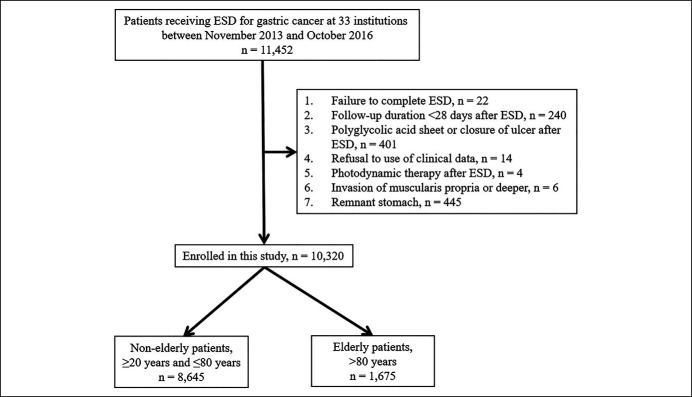
Workflow for patient enrollment to investigate the incidence of delayed bleeding after endoscopic submucosal dissection (ESD).

We enrolled a total of 11,452 patients with GC resected by ESD at 33 institutions (Figure 1). Of these, 1,132 patients met an exclusion criterion and were excluded, namely failure to complete ESD (n = 22), follow-up duration of <28 days after ESD (n = 240), PGA sheet or closure of ulcer after ESD (n = 401), refusal to the use of clinical data (n = 14), photodynamic therapy after ESD (n = 4), invasion of the muscularis propria or deeper (n = 6), and remnant stomach (n = 445). Finally, we analyzed the data of a total of 10,320 patients. No patient experienced cancellation of ESD or required surgery due to massive intraoperative bleeding during the ESD procedure.

Among clinical characteristics, the mean age was 71.7 ± 9.1 years, and the percentage of men was 74.2% (Table 1). Antithrombotic drugs were used by 18.0% of patients (1,860/10,320) and antiplatelet drugs and anticoagulants by 13.8% (1,428/10,320) and 5.6% (579/10,320) of patients, respectively. Regarding adverse events, the incidence of delayed bleeding and perforation was 4.7% and 1.5%, respectively (Table 1).

**Table 1.** Characteristics of nonelderly and elderly patients with gastric cancer receiving ESD

	Total patients (n = 10,320)	Nonelderly patients, younger than 80 yr (n = 8,645)	Elderly patients, older than 80 yr (n = 1,675)	*P*
Demographic				
Age, yr, mean ± SE	71.7 ± 9.1	69.3 ± 7.9	83.9 ± 2.6	<0.001
Men, n (%)	7,660 (74.2)	6,533 (75.6)	1,127 (67.3)	<0.001
Comorbidities				
Ischemic heart disease, n (%)	730 (7.1)	550 (6.4)	180 (10.7)	<0.001
Liver cirrhosis, n (%)	192 (1.9)	158 (1.8)	34 (2.0)	0.575
Hemodialysis, n (%)	155 (1.5)	115 (1.3)	40 (2.4)	0.001
AT therapy, n (%)	1,860 (18.0)	1,376 (15.9)	484 (28.9)	<0.001
APA, n (%)	1,428 (13.8)	1,041 (12.0)	387 (23.1)	<0.001
Aspirin, n (%)	981 (9.5)	722 (8.4)	259 (15.5)	<0.001
Cilostazol, n (%)	236 (2.3)	166 (1.9)	70 (4.2)	<0.001
Thienopyridine, n (%)	460 (4.5)	345 (4.0)	115 (6.9)	<0.001
Anticoagulant drug, n (%)	579 (5.6)	445 (5.1)	134 (8.0)	<0.001
Warfarin, n (%)	326 (3.2)	238 (2.8)	88 (5.3)	<0.001
DOAC, n (%)	253 (2.5)	207 (2.4)	46 (2.7)	0.394
Interruption of AT agents, n (%)	1,406 (13.6)	1,034 (12.0)	372 (22.2)	<0.001
One kind of AT agent, n (%)	1,215 (11.8)	887 (10.3)	328 (19.6)	<0.001
Two kinds of AT agent, n (%)	181 (1.8)	1,422 (1.6)	39 (2.3)	
Three kinds of AT agent, n (%)	10 (0.1)	5 (0.1)	5 (0.3)	
Replacement of APAs, n (%)	121 (1.2)	83 (1.0)	38 (2.3)	<0.001
Heparin bridging, n (%)	429 (4.2)	322 (3.7)	107 (6.4)	<0.001
Lesion				
Multiple tumors, n (%)	1,294 (12.5)	1,053 (12.2)	241 (14.4)	0.013
Location in lower third of stomach, n (%)	4,688 (45.4)	3,860 (44.7)	828 (49.4)	<0.001
Predominance of undifferentiated type, n (%)	506 (4.9)	469 (5.4)	37 (2.2)	<0.001
Tumor size, mm, mean ± SE	17.7 ± 12.5	17.4 ± 12.2	19.4 ± 13.9	<0.001
Invasion to SM2, n (%)	655 (6.3)	535 (6.2)	120 (7.2)	0.134
Ulceration (scar), n (%)	977 (9.5)	813 (9.4)	164 (9.8)	0.631
Procedure				
Procedure time >120 min, n (%)	1,886 (18.3)	1,570 (18.2)	316 (19.0)	0.453
En block resection, n (%)	10,261 (99.4)	8,599 (99.5)	1,662 (99.2)	0.225
Second-look endoscopy, n (%)	7,384 (71.6)	6,128 (70.9)	1,256 (75.0)	0.001
Use of antacid drug (PPI/P-CAB/H2RA), n (%)	10,303 (99.8)	8,632 (99.8)	1,671 (99.8)	0.414
Bleeding, n (%)	489 (4.7)	393 (4.5)	96 (5.7)	0.037
Perforation, n (%)	154 (1.5)	127 (1.2)	27 (1.6)	0.802
Hospital stay duration, d, mean ± SE	7.0 ± 4.0	6.9 ± 3.7	7.5 ± 5.2	<0.001

*P*: statistical difference between nonelderly and elderly patients.

APA, antiplatelet agent; AT, antithrombotic; DOAC, direct oral anticoagulant; ESD, endoscopic submucosal dissection; H2RA, histamine 2 receptor antagonist; P-CAB, potassium-competitive acid blocker; PPI, proton pump inhibitor; SM2, submucosal invasion ≥500 μm from the muscularis mucosa.

The incidence of bleeding after ESD was significantly higher in patients with comorbidities, use of antithrombotic drugs, replacement of an antithrombotic drug with aspirin or cilostazol, interruption of an antithrombotic drug, and heparin bridging (Table 2). Patients with multiple tumors, lesions located in the lower third of the stomach, and a tumor size >30 mm had a significantly increased incidence of bleeding. Among ESD-related factors, a procedure time of >120 minutes was associated with a significantly increased incidence of bleeding (Table 2). No patient required surgery or died due to delayed bleeding after ESD. Transfusion was performed in 1.4% (143/10,320) of all patients, 1.3% (111/8,645) of nonelderly patients, and 1.9% (32/1,675) of elderly patients (Table 2).

**Table 2.** Numbers and rates of delayed bleeding after ESD in nonelderly and elderly patients

	Total (n = 10,320)			Nonelderly patients, ≤80 yr (n = 8,645)			Elderly patients, >80 yr (n = 1,675)			*P*
	With factor	Without factor	P*	With factor	Without factor	P*	With factor	Without factor	P*	
Comorbidities										
Ischemic heart disease, n (%)	102/730 (14.0)	387/9,590 (4.0)	<0.001	84/550 (15.3)	309/8,095 (3.8)	0.001	18/180 (10.0)	78/1,495 (5.2)	0.016	0.119
Liver cirrhosis, n (%)	14/192 (7.3)	475/10,128 (4.7)	0.118	12/158 (7.6)	381/8,847 (4.5)	0.079	2/34 (5.9)	94/1,641 (5.7)	1.000	0.745
Hemodialysis, n (%)	36/155 (23.2)	453/10,165 (4.5)	<0.001	27/115 (23.5)	366/8,530 (4.3)	<0.001	9/40 (22.5)	87/1,635 (5.3)	<0.001	0.921
AT therapy, n (%)	222/1,860 (11.9)	267/8,460 (3.2)	<0.001	177/1,376 (12.9)	216/7,629 (3.0)	<0.001	45/484 (9.3)/	51/1,191 (4.3)	<0.001	0.063
APA, n (%)	155/1,428 (10.9)	334/8,892 (3.8)	<0.001	122/1,041 (11.7)	271/7,604 (3.6)	<0.001	33/387 (8.5)	63/1,288 (4.9)	0.009	0.120
Aspirin, n (%)	110/981 (11.2)	379/9,339 (4.1)	<0.001	88/722 (12.2)	305/7,923 (3.8)	<0.001	22/259 (8.5)	74/1,416 (5.2)	0.042	0.145
Cilostazol, n (%)	20/236 (8.5)	469/10,084 (4.7)	0.012	13/166 (7.8)	380/8,479 (4.5)	0.050	7/70 (10.0)	89/1,605 (5.5)	0.116	0.617
Thienopyridine, n (%)	67/460 (14.6)	422/9,860 (4.3)	<0.001	56/345 (16.2)	337/8,300 (4.1)	<0.001	11/115 (9.6)	85/1,560 (5.4)	0.091	0.124
Anticoagulant drug, n (%)	111/579 (19.2)	378/9,741 (3.9)	<0.001	91/445 (20.4)	302/8,200 (3.7)	<0.001	20/134 (14.9)	76/1,541 (4.9)	<0.001	0.235
Warfarin, n (%)	68/326 (20.9)	421/9,994 (4.2)	<0.001	52/238 (21.8)	341/8,407 (4.1)	<0.001	16/88 (18.2)	80/1,587 (5.0)	<0.001	0.556
DOAC, n (%)	43/253 (17.0)	446/10,067 (4.4)	<0.001	39/207 (18.8)	354/8,438 (4.2)	<0.001	4/46 (8.7)	92/1,629 (5.6)	0.332	0.151
Interruption of AT agents, n (%)	175/1,406 (12.4)	314/8,911 (3.5)	<0.001	140/1,034 (13.5)	253/7,608 (3.3)	<0.001	35/372 (9.4)	61/1,303 (4.7)	0.001	0.065
One kind of AT agent, n (%)	137/1,215 (11.3)	314/8,911 (3.5)	<0.001	108/887 (12.2)	253/7,608 (3.3)	<0.001	29/328 (8.8)	61/1,303 (4.7)	<0.001	0.142
Two kinds of AT agent, n (%)	34/181 (18.8)	314/8,911 (3.5)		29/142 (20.4)	253/7,608 (3.3)		5/39 (12.8)	61/1,303 (4.7)		
Three kinds of AT agent, n (%)	4/10 (40.0)	314/8,911 (3.5)		3/5 (60.0)	253/7,608 (3.3)		1/5 (20.0)/	61/1,303 (4.7)		
Replacement of APAs, n (%)	14/121 (11.6)	475/10,198 (4.7)	0.002	10/83 (12.0)	383/8,561 (4.5)	0.004	4/38 (10.5)	92/1,637 (5.6)	0.273	0.824
Heparin bridging, n (%)	77/429 (17.9)	412/9,891 (4.2)	<0.001	63/322 (19.6)	330/8,323 (4.0)	<0.001	14/107 (13.1)/	82/1,568 (5.2)	0.004	0.200
Lesion										
Multiple tumors, n (%)	83/1,294 (6.4)	406/9,026 (4.5)	0.003	63/1,053 (6.0)	330/7,592 (4.3)	0.022	20/241 (8.3)	76/1,434 (5.3)	0.072	0.218
Location in lower third of stomach, n (%)	270/4,688 (5.8)	219/5,632 (3.9)	<0.001	213/3,860 (5.5)	180/4,785 (3.8)	<0.001	57/828 (6.9)	39/847 (4.6)	0.046	0.150
Undifferentiated type, n (%)	26/506 (5.1)	463/9,814 (4.7)	0.677	22/469 (4.7)/	371/8,176 (4.5)	0.820	4/37 (10.8)	92/1,638 (5.6)	0.158	0.132
Tumor size (>30 mm), n (%)	84/1,217 (6.9)	405/9,103 (4.4)	<0.001	66/982 (6.7)	327/7,663 (4.3)	0.001	18/235 (7.7)	78/1,440 (5.4)	0.173	0.635
Invasion to SM2, n (%)	39/655 (6.0)	450/9,664 (4.7)	0.125	30/535 (5.6)	363/8,109 (4.5)	0.237	9/120 (7.5)/	87/1,555 (5.6)	0.411	0.458
Ulceration (scar), n (%)	51/977 (5.2)	437/9,320 (4.7)	0.479	39/813 (4.8)	353/7,811 (4.5)	0.723	12/164 (7.3)	84/1,508 (5.6)	0.375	0.212
Procedure										
Procedure time >120 min, n (%)	112/1,886 (5.9)	375/8,411 (4.5)	0.007	90/1,570 (5.7)	302/7,061 (4.3)	0.016	22/316 (7.0)	73/1,350 (5.4)	0.282	0.428
En block resection, n (%)	486/10,259 (4.7)	3/61 (4.9)	0.765	391/8,597 (4.5)	2/48 (4.2)	0.899	95/1,662 (5.7)	1/13 (7.7)	0.537	0.051
Second-look endoscopy, n (%)	361/7,384 (4.9)	128/2,936 (4.4)	0.259	292/6,128 (4.8)	101/2,517 (4.0)	0.139	69/1,256 (5.5)	27/419 (6.4)	0.468	0.300
Perforation, n (%)	5/154 (3.2)	483/10,149 (4.8)	0.664	4/127 (3.1)	389/8,503 (4.6)	0.522	1/27 (3.7)/	94/1,646 (5.7)	0.192	0.887
Transfusion, n (%)	138/143 (96.5)	350/10,176 (3.4)	<0.001	107/111 (96.4)	285/8,533 (3.3)	<0.001	31/32 (96.9)	65/1,643 (4.0)	<0.001	0.986
Surgery, n (%)	0 (0)	489/10,320 (4.7)	—	0 (0)	393/8,645 (4.5)	—	0 (0)	96/1,675 (5.7)	—	—
Death, n (%)	0 (0)	489/10,320 (4.7)	—	0 (0)	393/8,645 (4.5)	—	0 (0)	96/1,675 (5.7)	—	—

*P*: comparison of the rate of patients with the factor between nonelderly patients and elderly patients.

P*: comparison of the rate between patients with and without the factor.

APA, antiplatelet agent; AT, antithrombotic; DOAC, direct oral anticoagulant; ESD, endoscopic submucosal dissection; H2RA, histamine 2 receptor antagonist; P-CAB, potassium-competitive acid blocker; PPI, proton pump inhibitor; SM2, submucosal invasion ≥500 μm from the muscularis mucosa.

### GC patients receiving ESD between nonelderly and elderly populations

Although the World Health Organization defines people aged 65–74 years as early-stage elderly, we divided patients into 2 groups: nonelderly patients aged older than 20 years and younger than 80 years (n = 8,645) and elderly patients older than 80 years (n = 1,675). Rates of patients with ischemic heart disease and hemodialysis and use of antithrombotic drugs, antiplatelet drugs (aspirin, cilostazol, and thienopyridine), and anticoagulants (warfarin) were significantly higher in the elderly than those in the nonelderly patients (Table 1). Lesion characteristics, such as location in the stomach, differentiated type of tumor, and tumor size, were significantly different between the 2 groups, whereas procedure-related factors, such as procedure time and en block resection rate, were similar. Among adverse events, the incidence of bleeding in the elderly patients was 5.7%, which was significantly higher than that in the nonelderly patients (4.5%) (*P* = 0.037) (Table 1).

In both nonelderly and elderly patients, the incidence of bleeding was significantly higher in men; patients with ischemic heart disease and hemodialysis; those receiving antiplatelet drugs, aspirin, anticoagulants, or warfarin or in whom an antithrombotic drug was replaced with aspirin or cilostazol; and those who underwent heparin bridging (Table 2). Among lesion-related factors, a lesion location in the lower third of the stomach was associated with a significantly increased incidence of bleeding in both nonelderly and elderly patients. Although the factors related to a high incidence of bleeding in the elderly and nonelderly patients were similar, the nonelderly patients had a higher incidence of these factors than the elderly patients.

Predictive factors showing a *P* value <0.05 in univariate analysis were included in multivariate logistic regression analysis. For ESD-associated bleeding at the time of treatment in nonelderly patients aged older than 20 and younger than 80 years, risk was increased in patients with ischemic heart disease (odds ratio [OR]: 1.861, 95% confidence interval [CI]: 1.295–2.673), hemodialysis (4.363, 2.652–7.178), aspirin (1.898, 1.332–2.702), thienopyridine (3.205, 2.012–5.105), warfarin (6.945, 3.951–12.209), and direct oral anticoagulant (DOAC) (7.934, 4.692–13.414) use, tumor size >30 mm (1.763, 1.322–2.350), and tumor location in the lower third (1.597, 1.287–1.981) (Table 3).

**Table 3.** Univariate and multivariate logistic regression analysis of predictive factors for ESD-associated bleeding at the time of treatment in nonelderly patients

		Univariate analysis			Multivariate analysis		
		OR	95% CI	*P*	OR	95% CI	*P*
Sex	Men	1.633	1.248–2.138	<0.001	1.258	0.951–1.663	0.108
Ischemic heart disease	Yes	4.542	3.507–5.883	<0.001	1.861	1.295–2.673	0.001
Liver cirrhosis	Yes	1.749	0.962–3.178	0.067			
Hemodialysis	Yes	6.844	4.391–10.666	<0.001	4.363	2.652–7.178	<0.001
Aspirin	Yes	3.467	2.698–4.454	<0.001	1.898	1.332–2.702	<0.001
Cilostazol	Yes	1.811	1.019–3.220	0.043	1.400	0.736–2.663	0.305
Thienopyridine	Yes	4.579	3.371–6.219	<0.001	3.205	2.012–5.105	<0.001
Warfarin	Yes	6.613	4.773–9.162	<0.001	6.945	3.951–12.209	<0.001
DOAC	Yes	5.301	3.683–7.631	<0.001	7.934	4.692–13.414	<0.001
Interruption of AT agents	Yes	4.553	3.661–5.660	<0.001	0.709	0.454–1.107	0.130
Replacement of APAs	Yes	2.925	1.499–5.709	0.002	0.760	0.350–1.651	0.488
Heparin bridging	Yes	5.892	4.380–7.925	<0.001	1.011	0.622–1.643	0.965
Number of tumors	Multiple	1.400	1.061–1.848	0.017	1.251	0.932–1.681	0.136
Tumor size	>30 mm	1.616	1.230–2.124	0.001	1.763	1.322–2.350	<0.001
Tumor location	Lower third	1.494	1.219–1.831	<0.001	1.597	1.287–1.981	<0.001
Tumor differentiation	Undifferentiated	1.035	0.667–1.609	0.877			

APA, antiplatelet agent; AT, antithrombotic; CI, confidence interval; DOAC, direct oral anticoagulant; ESD, endoscopic submucosal dissection; OR, odds ratio.

In univariate analysis in elderly patients older than 80 years, predictive factors for bleeding after ESD were ischemic heart disease (OR: 2.019, 95% CI: 1.179–3.456), hemodialysis (5.166, 2.385–11.189), aspirin (1.683, 1.026–2.763) and warfarin (4.186, 2.329–7.526) use, heparin bridging (2.727, 1.491–4.992), and tumor location (1.532, 1.007–2.329) (Table 4). On multivariate logistic regression analysis in elderly patients, the only risk factors were hemodialysis (OR: 4.591, 95% CI: 2.056–10.248, *P* < 0.001) and warfarin use (OR: 4.783, 95% CI: 1.689–13.540, *P* = 0.003) (Table 4).

**Table 4.** Univariate and multivariate logistic regression analysis of predictive factors for ESD-associated bleeding at the time of treatment in elderly patients

		Univariate analysis			Multivariate analysis		
		OR	95% CI	*P*	OR	95% CI	*P*
Sex	Men	1.330	0.838–2.111	0.227			
Ischemic heart disease	Yes	2.019	1.179–3.456	0.010	1.422	0.735–2.751	0.296
Liver cirrhosis	Yes	1.029	0.243–4.357	0.969			
Hemodialysis	Yes	5.166	2.385–11.189	<0.001	4.591	2.056–10.248	<0.001
Aspirin	Yes	1.683	1.026–2.763	0.039	1.370	0.753–2.492	0.303
Cilostazol	Yes	1.893	0.842–4.253	0.122			
Thienopyridine	Yes	1.835	0.950–3.547	0.071			
Warfarin	Yes	4.186	2.329–7.526	<0.001	4.783	1.689–13.540	0.003
DOAC	Yes	1.591	0.558–4.522	0.385			
Interruption of AT agents	Yes	1.503	0.909–2.484	0.112			
Replacement of APAs	Yes	1.976	0.686–5.686	0.207			
Heparin bridging	Yes	2.727	1.491–4.992	0.001	0.755	0.257–2.217	0.609
Number of tumors	Multiple	1.617	0.968–2.700	0.168			
Tumor size	>30 mm	1.448	0.851–2.466	0.172			
Tumor location	Lower third	1.532	1.007–2.329	0.046	1.408	0.918–2.161	0.117
Tumor differentiation	Undifferentiated	2.037	0.707–5.872	0.188			

APA, antiplatelet agent; AT, antithrombotic; CI, confidence interval; DOAC, direct oral anticoagulant; ESD, endoscopic submucosal dissection; OR, odds ratio.

Hospital stay duration in nonelderly and elderly patients with delayed bleeding after ESD was 10.4 ± 7.0 days and 10.4 ± 6.1 days, whereas that in nonelderly and elderly patients without delayed bleeding was 6.8 ± 3.7 days and 7.3 ± 5.1 days, respectively. There was no significant difference of hospital stay duration between nonelderly and elderly patients.

### Predictive factors for ESD-associated bleeding in different generations

To evaluate the incidence of bleeding after ESD in different generations and to clarify risk factors at the time of treatment, we divided patients into 4 groups: 20–40 years, 40–60 years, 60–80 years, and older than 80 years (see Supplementary Tables 1 and 2, Supplementary Digital Content 1, http://links.lww.com/CTG/A690). Regarding characteristics of GC, the rate of the undifferentiated type in patients aged 20–40 years was 53.2%. The use of antithrombotic drugs in patients aged 60–80 years and older than 80 years was higher than in younger generations. On univariate analysis, although risk for bleeding was significantly increased in the patients aged 40–60 years and DOAC use, no significant association with other drugs was seen (see Supplemental Table 2, Supplementary Digital Content 1, http://links.lww.com/CTG/A690).

## DISCUSSION

In elderly patients older than 80 years, judgment of whether medication and treatment will improve prognosis requires careful consideration of the individual's condition. Because the number of GC patients older than 80 years has recently increased in Japan with the aging of society, evaluation of the characteristics of elderly GC patients and their incidence of ESD-related adverse events is critical to identifying risk factors for adverse events. Generally, ESD is technically demanding and may be associated with severe complications. It requires training and experience with a positive attitude to learning that improves skill and minimizes unwanted complications. This study was a retrospective multicenter observational study that included more than 10,000 patients undergoing ESD from Japanese endoscopists who were experienced and skilled in this procedure. The incidence of bleeding after ESD in patients older than 80 years of 5.7% (95% CI: 4.6%–6.9%) was significantly higher than the 4.5% (95% CI: 4.1%–5.0%) in patients younger than 80 years. Patients older than 80 years have a wider range of possible factors associated with bleeding after ESD than those younger than 80 years, including hemodialysis, use of antiplatelet drugs, aspirin, thienopyridine, anticoagulant drugs, and warfarin, multiple tumors, tumor size >30 mm, and tumor location in the lower third of the stomach (Table 1). Accordingly, they require attention to the possibility of adverse events, particularly those receiving hemodialysis and taking warfarin.

### Incidence of delayed bleeding after ESD in elderly patients

GC is the third most common cancer diagnosed worldwide in men and the third leading cause of cancer-related death in men, affecting approximately 1 million new individuals each year and causing at least 700,000 deaths (15,16). National GC screening programs using endoscopy in Korea and Japan may have contributed to a decrease in GC mortality by decreasing diagnosis at an advanced stage and by *Helicobacter pylori* eradication therapy after endoscopy (17). In Japan, although approximately 50,000 GC deaths occurred annually over the past 40 years, deaths have lately significantly decreased, from 50,136 in 2010 to 42,931 in 2019, after the expansion of insurance coverage to include eradication therapy (18,19). However, given the increased life expectancy of Japanese, the number of GC deaths in Japanese patients aged 80 years or older and with GC has also increased, from 19,983 in 2010 to 21,284 in 2019 (19). In patients older than 80 years, although endoscopic and surgical procedures for GC were previously often avoided because of age, severe comorbidities, and low activities of daily living scores, recent improvement in life expectancy has increased the number of patients suitable for such procedures.

Among adverse events, reported rates of bleeding after ESD for GC range from 4.1% to 8.5% of patients (8,9,20,21). A recent meta-analysis reported a pooled rate of bleeding of 5.1% (95% CI: 4.5%–5.7%) (22) and suggested a number of possible factors, including male sex, cardiopathy, antithrombotic drugs, liver cirrhosis, chronic kidney disease, tumor size > 20 mm, resected specimen size > 30 mm, location in the lesser curvature, flat/depressed type, histology, and ulceration. This meta-analysis also reported that a patient’s age of 75 years or older is not a risk factor for bleeding (22). Watanabe et al. (23) reported that there was no significant difference in the incidence of bleeding among different age groups (patients aged 85 years or older [4.2%], patients aged 65–84 years [4.8%], and patients aged 64 years or younger [6.2%]), as was also observed in other reports of patients aged ≥75 years (24–27). These reports had small sample sizes, however, leaving 2 questions unanswered, namely whether the incidence of bleeding after ESD was similar between the nonelderly and elderly populations and what risk factors were characteristic for bleeding in elderly patients undergoing ESD. In this multicenter study, the rate of bleeding in elderly patients older than 80 years was 5.7% (96/1,675), which was significantly higher than that in nonelderly patients (4.5%, 393/8,645). Considered together with 3 additional factors—the incidence of bleeding in patients older than 80 years was not 2–3 times greater than that in nonelderly patients; the higher incidence of general possible risk factors for bleeding in patients older than 80 years, such as use of antithrombotic drugs; and the fact that patients without such risk factors also had high rate of bleeding—our findings indicate the need for more careful management of ESD to prevent bleeding in patients older than 80 years.

In this study, although we excluded 401 patients having use of a PGA sheet or clip closure of ulcers after ESD (3.5%), these practices are no longer common (28,29). However, clip closure is common after endoscopic mucosal resection, and the use of endoscopic closure after ESD has been reported to prevent delayed bleeding (30). In addition, the tissue-shielding method using a PGA sheet is also used to prevent delayed bleeding after ESD in patients at high risk for delayed bleeding (31). Therefore, further study to clarify the efficacy of ulcer closure and tissue-shielding method should include these patients.

### Risk factors of delayed bleeding after ESD in elderly patients

Risks of bleeding after ESD can be classified into several categories, including patient-related, medication-related, lesion-related and procedure-related factors, and acid inhibitory drugs (6,22,32). Accurate identification of risks among these categories can help to guide management after ESD, especially in patients with high risk. We previously developed a prediction model (Bleeding after Endoscopic Submucosal dissection Trend from Japan [BEST-J] score) for bleeding after ESD for GC, which was derived from a combination of 10 variables (12). Rates of bleeding at low (0–1 points), intermediate (2 points), high (3–4 points), and very high risk (≥5 points) were 2.8%, 6.1%, 11.4%, and 29.7%, respectively (12). In this study, when we divided patients into nonelderly and elderly patients, aged younger than 80 and older than 80 years, respectively, most of the risk factors for bleeding were similar between the 2 groups, and no specific new risk factors were identified (22). In addition, surprisingly, although the rate of bleeding in patients older than 80 years was significantly higher than that in patients younger than 80 years, major risks (e.g., antithrombotic drugs, DOAC, heparin bridging, and location and size of lesion) did not increase the risk of bleeding. A prediction model using the BEST-J score in patients aged 85 years or older showed modest discrimination, with a *c* statistic of 0.65 (95% CI: 0.55–0.75), which was lower than that in the nonelderly population. This may be because the rate of bleeding in patients without major risks is relatively high. A follow-on study should investigate whether the BEST-J score should be selected as a prediction model for bleeding after ESD for GC in patients older than 80 years.

### Limitations

This study has several limitations. First, it was conducted under a retrospective design. Second, all patients were Japanese, and it is unclear whether elderly patients in other populations have similar characteristics for bleeding. Third, we did not analyze survival rates in patients with early-stage GC between those who underwent ESD and those who did not. Further prospective studies are necessary to confirm the safety and efficacy of ESD for GC in patients older than 80 years.

### Conclusions

ESD is less invasive than open surgery and improves quality of life in patients with early-stage GC. In this nationwide multicenter study in elderly Japanese older than 80 years, the incidence of bleeding after ESD was high, especially in patients receiving hemodialysis and taking warfarin.

## CONFLICTS OF INTEREST

**Guarantor of the article:** Mitsushige Sugimoto, MD, PhD.

**Author contributors:** Conception and design: M.S. and M.M.; acquisition of data: M.S., W.H., Y.T., S.S., N.K., S.H., H.D., Y.N., T.H., M.K., Y.M., T.S., M.I., H.T., T.I., T.M., K.H., A.G., T.M., H.N., D.N., K.O., H.U., Y.H., M.M., S.Y., T.M., T.Y., Y.A., T.N., S.K., S.Ku., K.M., and T.K.; analysis and interpretation of data: M.S. and M.M.; drafting of the manuscript: MS and MM; critical revision of the manuscript: Y.T., M.F., T.K., H.S., and A.M.; statistical analysis: M.S. and M.M.; and study supervision: M.F., A.M., and T.K. All authors listed contributed substantially to the design, data collection and analysis, and editing of the manuscript.

**Financial support:** None to report.

**Potential competing interests:** M.F. declares that he has received lecture honoraria from Takeda Pharmaceutical, EA Pharma, and Nihon Pharmaceutical and that his department has received research grants from HOYA Pentax, EA Pharma, Eisai, Taiho Pharmaceutical, AbbVie GK, Nippon Kayaku, Chugai Pharmaceutical, Gilead Sciences, Kyorin Pharmaceutical, and Mitsubishi Tanabe Pharma outside the submitted work.

**Study Highlights**

**WHAT IS KNOWN**

✓ Elderly patients are generally at a higher risk of death from a range of causes, such as adverse events after endoscopic treatment.
✓ Little is known about the incidence rate and characteristic risk factors for delayed bleeding in elderly patients undergoing endoscopic submucosal dissection.

**WHAT IS NEW HERE**

✓ he incidence of delayed bleeding in elderly patients older than 80 years was significantly higher than that in nonelderly (older than 20 years and younger than ≤80 years) patients.
✓ Compared with nonelderly patients, elderly patients had a higher rate of various possible factors for bleeding after endoscopic submucosal dissection, such as antiplatelet drugs, anticoagulants, multiple tumors, tumor size >30 mm, and tumor location.
✓ On multivariate analysis, the risk factors in elderly patients were hemodialysis and warfarin use.

## Supplementary Material

SUPPLEMENTARY MATERIAL
