# Supplementary material for: Risk Factors for Bleeding After Endoscopic Submucosal Dissection for Gastric Cancer in Elderly Patients Older Than 80 Years in Japan
Source: Clin Transl Gastroenterol. 2021 Sep 24;12(9):e00404. doi: 10.14309/ctg.0000000000000404 (PMC8659993; doi:10.14309/ctg.0000000000000404)
Supplement: SUPPLEMENTARY MATERIAL [file ct9-12-e00404-s002.docx]

Supplemental Table 1. Characteristics of patients with gastric cancer receiving ESD in different generations

|  | >20 years,  <40 years  (n = 47) | >40 years,  <60 years  (n = 1,009) | >60 years,  <80 years  (n = 7,589) | >80 years  (n = 1,675) |
| --- | --- | --- | --- | --- |
| Demographic |  |  |  |  |
| Age (years), mean ± SE | 37.5 ± 2.4 | 54.6 ± 4.9 | 71.5 ± 5.3 | 83.9 ± 2.6 |
| Male gender, n (%) | 29 (61.7%) | 747 (74.0%) | 5757 (75.9%) | 1,127 (67.3%) |
| Comorbidities |  |  |  |  |
| Ischemic heart disease, n (%) | 0 (0%) | 21 (2.1%) | 529 (7.0%) | 180 (10.7%) |
| Liver cirrhosis, n (%) | 0 (0%) | 10 (1.0%) | 148 (2.0%) | 34 (2.0%) |
| Hemodialysis, n (%) | 0 (0%) | 9 (0.9%) | 106 (1.4%) | 40 (2.4%) |
| AT therapy, n (%) | 0 (0%) | 36 (3.6%) | 1,340 (17.7%) | 484 (28.9%) |
| APA, n (%) | 0 (0%) | 31 (3.1%) | 1,010 (13.3%) | 387 (23.1%) |
| Aspirin, n (%) | 0 (0%) | 21 (2.1%) | 701 (9.2%) | 259 (15.5%) |
| Cilostazol, n (%) | 0 (0%) | 3 (0.3%) | 163 (2.1%) | 70 (4.2%) |
| Thienopyridine, n (%) | 0 (0%) | 11 (1.1%) | 334 (4.4%) | 115 (6.9%) |
| Anticoagulant drug, n (%) | 0 (0%) | 9 (0.9%) | 436 (5.7%) | 134 (8.0%) |
| Warfarin, n (%) | 0 (0%) | 6 (0.6%) | 232 (3.1%) | 88 (5.3%) |
| DOAC, n (%) | 0 (0%) | 3 (0.3%) | 204 (2.7%) | 46 (2.7%) |
| Interruption of AT agents, n (%) | 0 (0%) | 30 (3.0%) | 1004 (13.2%) | 372 (22.2%) |
| Replacement of APAs, n (%) | 0 (0%) | 2 (0.2%) | 81 (1.1%) | 38 (2.3%) |
| Heparin bridging, n (%) | 0 (0%) | 5 (0.5%) | 317 (4.2%) | 107 (6.4%) |
| Lesion |  |  |  |  |
| Multiple tumors, n (%) | 3 (6.4%) | 58 (5.7%) | 992 (13.1%) | 241 (14.4%) |
| Location in lower third of stomach, n (%) | 25 (53.2%) | 488 (44.4%) | 3387 (44.6%) | 828 (49.4%) |
| Predominance of undifferentiated type, n (%) | 27 (57.4%) | 171 (16.9%) | 271 (3.6%) | 37 (2.2%) |
| Tumor size (mm), mean ± SE | 17.6 ± 13.7 | 16.2 ± 11.2 | 17.5 ± 12.3 | 19.4 ± 13.9 |
| Invasion to SM2, n (%) | 2 (4.3%) | 57 (5.6%) | 476 (6.3%) | 120 (7.2%) |
| Ulceration (scar), n (%) | 6 (12.8%) | 114 (11.3%) | 693 (9.2%) | 164 (9.8%) |
| Procedure |  |  |  |  |
| Procedure time >120 min, n (%) | 9 (19.1%) | 179 (17.7%) | 1382 (18.2%) | 316 (19.0%) |
| En block resection, n (%) | 47 (100%) | 1006 (99.7%) | 7544 (99.4%) | 1662 (99.2%) |
| Second-look endoscopy, n (%) | 30 (63.8%) | 687 (68.1%) | 5411 (71.3%) | 1256 (75.0%) |
| Bleeding, n (%) | 5 (10.6%) | 33 (3.3%) | 355 (4.7%) | 96 (5.7%) |
| Perforation, n (%) | 3 (6.4%) | 20 (2.0%) | 104 (1.4%) | 27 (1.6%) |
| Hospital stay duration (day), mean ± SE | 6.3 ± 2.3 | 6.6 ± 2.7 | 7.0 ± 3.8 | 7.5 ± 5.2 |

Abbreviations: APA, antiplatelet agent; AT, antithrombotic; DOAC, direct oral anticoagulant; ESD: endoscopic submucosal dissection, SM2, submucosal invasion ≥500 μm from the muscularis mucosa

Supple Table 2. Univariate analysis of predictive factors for ESD-associated bleeding at the time of treatment in different generations

|  |  | >20 years, <40 years  (n = 47) | | | >40 years, <60 years  (n = 1,009) | | | >60 years, <80 years  (n = 7,589) | | | >80 years  (n = 1,675) | | |
| --- | --- | --- | --- | --- | --- | --- | --- | --- | --- | --- | --- | --- | --- |
|  |  | OR | 95% CI | P value | OR | 95% CI | P value | OR | 95% CI | P value | OR | 95% CI | P value |
| Sex | Male | 0.923 | 0.139-6.138 | 0.934 | 1.314 | 0.563-3.063 | 0.528 | 1.678 | 1.260-2.234 | <0.001 | 1.330 | 0.838-2.111 | 0.227 |
| Ischemic heart disease | Yes | - |  |  | 7.781 | 2.463-24.577 | <0.001 | 4.366 | 3.345-5.799 | <0.001 | 2.019 | 1.179-3.456 | 0.010 |
| Liver cirrhosis | Yes | - |  |  | - |  |  | 1.816 | 0.997-3.309 | 0.051 | 1.029 | 0.243-4.357 | 0.969 |
| Hemodialysis | Yes | - |  |  | 16.167 | 3.858-67.741 | <0.001 | 6.289 | 3.939-10.041 | <0.001 | 5.166 | 2.385-11.189 | <0.001 |
| Aspirin | Yes | - |  |  | 3.250 | 0.725-14.568 | 0.124 | 3.417 | 2.645-4.415 | <0.001 | 1.683 | 1.026-2.763 | 0.039 |
| Cilostazol | Yes | - |  |  | - |  |  | 1.785 | 1.003-3.179 | 0.049 | 1.893 | 0.842-4.253 | 0.122 |
| Thienopyridine | Yes | - |  |  | 3.019 | 0.375-24.299 | 0.299 | 4.542 | 3.327-6.200 | <0.001 | 1.835 | 0.950-3.547 | 0.071 |
| Warfarin | Yes | - |  |  | - |  |  | 6.684 | 4.809-9.290 | <0.001 | 4.186 | 2.329-7.526 | <0.001 |
| DOAC | Yes | - |  |  | 15.219 | 1.345-172.203 | 0.028 | 5.074 | 3.505-7.345 | <0.001 | 1.591 | 0.558-4.522 | 0.385 |
| Interruption of AT agents | Yes | - |  |  | 2.184 | 0.498-9.581 | 0.300 | 4.634 | 3.702-5.800 | <0.001 | 1.503 | 0.909-2.484 | 0.112 |
| Replacement of APAs | Yes | - |  |  | 30.469 | 1.864-498.09 | 0.017 | 2.573 | 1.276-5.188 | 0.008 | 1.976 | 0.686-5.686 | 0.207 |
| Heparin bridging | Yes | - |  |  | - |  |  | 5.891 | 4.366-7.948 | <0.001 | 2.727 | 1.491-4.992 | 0.001 |
| Number of tumors | Multiple | - |  |  | 1.675 | 0.496-5.658 | 0.407 | 1.367 | 1.027-1.819 | 0.032 | 1.617 | 0.968-2.700 | 0.168 |
| Tumor size | >30 mm | 6.333 | 0.803-49.921 | 0.080 | 0.973 | 0.291-3.249 | 0.964 | 1.610 | 1.211-2.142 | 0.001 | 1.448 | 0.851-2.466 | 0.172 |
| Tumor location | Lower third | 4.000 | 0.412-38.844 | 0.232 | 2.582 | 1.238-5.384 | 0.011 | 1.417 | 1.145-1.753 | 0.001 | 1.532 | 1.007-2.329 | 0.046 |
| Tumor differentiation | Undifferentiated | 10.250 | 0.533-196.98 | 0.123 | 1.080 | 0.252-4.631 | 0.917 | 1.237 | 0.826-1.852 | 0.302 | 2.037 | 0.707-5.872 | 0.188 |

Abbreviations: APA, antiplatelet agent; AT, antithrombotic; DOAC, direct oral anticoagulant; ESD: endoscopic submucosal dissection; CI: confidence interval; OR: odds ratio
